# The status of the genus Bostryx Troschel, 1847, with description of a new subfamily (Mollusca, Gastropoda, Bulimulidae)

**DOI:** 10.3897/zookeys.216.3646

**Published:** 2012-08-21

**Authors:** Abraham S.H. Breure

**Affiliations:** 1 Netherlands Centre for Biodiversity Naturalis, P.O. Box 9517, 2300 RA Leiden, the Netherlands

**Keywords:** Orthalicoidea, taxonomy, Bostrycinae subfam. n.

## Abstract

The status of the genus *Bostryx* is discussed and, based on morphological and molecular data, restricted to a group of species related to *Bostryx solutus*, for which the new subfamily name Bostrycinae is introduced.

## Introduction

Troschel (1847: 49) described a new, peculiar land snail, as *Bulimus (Bostryx) solutus*. He wrote: “Diese durch Herrn Dr. von Tschudi in Peru in vielen Exemplaren gesammelte Art ist so eigenthümlich, dass ich überzeugt bin, sie werde bei einer naturgemässen Theilung der Gattung *Bulimus*, wovon die Notwendigkeit nach meinem anatomischen Untersuchungen keinen Zweifel unterliegt, eine eigene Gattung bilden, für die ich den Namen *Bostryx* vorschlage”. However, Troschel’s conviction that *Bostryx* constituted a separate genus was not readily accepted. Most authors (e.g. Pilsbry 1896 [1895–1896], Thiele 1931) regarded it as a subgenus of *Bulimulus* Leach, 1814. It was not until 1944 when Pilsbry used it as a separate genus (Pilsbry 1944). Subsequent authors have classified many other taxa as subgenera — based purely on shell shape — within *Bostryx* (e.g. Zilch 1960 [1959–1960], Schileyko 1999). Breure (1979) in his revision of the Bulimulidae, using the external shell morphology and internal anatomical characters, listed 22 taxa as synonyms of *Bostryx* (*s.l*.)and 274 available names at the species-level. He wrote: “It has been mentioned before that a number of species groups may be recognized with *Bostryx* (sensu lato) that correspond more or less with some of the ‘subgenera’ listed in the above-mentioned synonymy. There are, however, a rather large number of taxa that can not be allocated to one of these species groups and it is preferred, therefore, to treat the genus here sensu lato”. During recent molecular work 10 *Bostryx* species were sequenced, showing that *Bostryx* (*s.l.*) is a polyphyletic taxon (Breure and Romero 2012). For the monophyletic species group with *Bostryx solutus* the subfamily name Bostrycinae subfam. n. was introduced; however, without proper diagnosis fulfilling the requirement of Art. 13.1 ICZN this is a nomen nudum. To correct this (surprising) mistake, the necessary data for a valid description are presented in this paper.

## Systematics

**Superfamily Orthalicoidea Martens in Albers, 1860**

**Family Bulimulidae Tryon, 1867**

### Subfamily Bostrycinae subfam. n.

urn:lsid:zoobank.org:act:66E52BE3-12DE-41B4-8455-1FAC18112985

#### Diagnosis.

Shells with a smooth protoconch; genital organs with a relatively long penis sheath (ca. 1/4–1/6 total phallus length) (Breure 1978: fig. 176).

#### Type genus.

*Bostryx* Troschel, 1847; type species by monotypy *Bulimus (Bostryx) solutus* Troschel, 1847.

#### Remarks.

It should be stressed that this genus needs a thorough revision, based both on a re-evaluation of morphological characters and molecular data. In molecular analyses, species of this subfamily are forming a monophyletic group with *Bostryx solutus* (Troschel, 1847) included. Given current understanding (Breure and Romero 2012, Breure unpublished data) the following, additional taxa at least belong to *Bostryx* *s.str.*: *Bostryx (Peronaeus) agueroi* Weyrauch, 1960, *Bostryx (Bostryx) aguilari* Weyrauch, 1967, *Bostryx (Bostryx) agueroi beltrani* Weyrauch, 1964, *Bulinus conspersus* Sowerby I, 1833, *Bostryx edmundi* Breure & Neubert, 2008, *Bostryx granulatus* Breure & Neubert, 2008, *Bostryx (Pseudoperonaeus) longispira* Weyrauch, 1960, *Bulinus modestus* Broderip, 1832, *Bostryx multiconspectus* Breure, 2008, *Drymaeus torallyi peruvianus* Pilsbry, 1944, *Bostryx primigenius* Breure, 2008, *Bulinus scalariformis* Broderip, 1832, *Helix sordidus* Lesson, 1826, *Bostryx (Multifasciatus) superbus* Weyrauch, 1967, *Helix torallyi* d’Orbigny, 1835.

As may be seen from this — necessarily incomplete — list, shell shape alone may be a misleading character for classification (e.g., with representatives of three ‘subgenera’). Further research needs to clarify which of the 22 synonyms of *Bostryx* *(s.l.)* may be given (sub)generic status within this subfamily.

Finally, Breure and Romero (2012) showed that *Helix apodemeta* d’Orbigny, 1835, *Bulimulus (Scansicochlea) strobeli* Parodiz, 1956, and *Bulinus bilineatus* Sowerby I, 1833 may need to be re-classified with *Naesiotus* Albers, 1850; this genus (also treated *s.l.* in Breure 1979) is in need of an in-depth revision and its relationship to *Bulimulus* to be clarified.

Further research, especially using molecular data, will undoubtedly give new insights, thus leading to either more support for the current classification or perhaps other surprises.

## Supplementary Material

XML Treatment for Bostrycinae
